# Retrospective Analysis of Bacterial and Viral Co-Infections in *Pneumocystis* spp. Positive Lung Samples of Austrian Pigs with Pneumonia

**DOI:** 10.1371/journal.pone.0158479

**Published:** 2016-07-18

**Authors:** Christiane Weissenbacher-Lang, Branislav Kureljušić, Nora Nedorost, Bettina Matula, Wolfgang Schießl, Daniela Stixenberger, Herbert Weissenböck

**Affiliations:** 1 Institute of Pathology and Forensic Veterinary Medicine, Department of Pathobiology, University of Veterinary Medicine Vienna, Vienna, Austria; 2 Department of Pathology, Institute of Veterinary Medicine of Serbia, Belgrade, Serbia; The University of Melbourne, AUSTRALIA

## Abstract

Aim of this study was the retrospective investigation of viral (porcine circovirus type 2 (PCV2), porcine reproductive and respiratory syndrome virus (PRRSV), torque teno sus virus type 1 and 2 (TTSuV1, TTSuV2)) and bacterial (*Bordetella bronchiseptica* (*B*. *b*.), *Mycoplasma hyopneumoniae* (*M*. *h*.), and *Pasteurella multocida* (*P*. *m*.)) co-infections in 110 *Pneumocystis* spp. positive lung samples of Austrian pigs with pneumonia. Fifty-one % were positive for PCV2, 7% for PRRSV, 22% for TTSuV1, 48% for TTSuV2, 6% for *B*. *b*., 29% for *M*. *h*., and 21% for *P*. *m*. In 38.2% only viral, in 3.6% only bacterial and in 40.0% both, viral and bacterial pathogens were detected. In 29.1% of the cases a co-infection with 1 pathogen, in 28.2% with 2, in 17.3% with 3, and in 7.3% with 4 different infectious agents were observed. The exposure to *Pneumocystis* significantly decreased the risk of a co-infection with PRRSV in weaning piglets; all other odds ratios were not significant. Four categories of results were compared: I = *P*. spp. + only viral co-infectants, II = *P*. spp. + both viral and bacterial co-infectants, III = *P*. spp. + only bacterial co-infectants, and IV = *P*. spp. single infection. The evaluation of all samples and the age class of the weaning piglets resulted in a predomination of the categories I and II. In contrast, the suckling piglets showed more samples of category I and IV. In the group of fattening pigs, category II predominated. Suckling piglets can be infected with *P*. spp. early in life. With increasing age this single infections can be complicated by co-infections with other respiratory diseases.

## Introduction

Respiratory diseases in pigs can be caused by a broad variety of pathogens and are mainly polymicrobial and multifactorial. The involved pathogens can strongly vary among production sites and, in the past, have been referred to as porcine respiratory disease complex (PRDC). This high diversity of pathogen combinations often renders a thorough diagnostic investigation as well as the establishment of generalized control or treatment programs difficult [1]. The relevant infectious agents involved in PRDC are present in sow herds generally with a low impact on the health status of the sows. Nevertheless, sows constitute a reservoir for the continuous circulation of respiratory pathogens in farrow-to-finish herds [2]. Due to the presence or absence of their capability of inducing lesions in respiratory tissue as a result of their own virulence, the respective causative organisms can be divided into primary and secondary or opportunistic infectious agents [3]. Besides non-infectious factors, currently only viral, bacterial and parasitic agents are considered. The impact of fungi has not yet been investigated. *Pneumocystis* spp. (*P*. spp.) belong to the opportunistic fungi and are of high clinical relevance in immunocompromised patients who due to massive proliferation of the fungus develop severe interstitial pneumonia [4]. As a group of strongly diversified organisms *P*. spp. are not exclusively adapted to humans. Various mammals, thereunder pigs, can also be infected [5–7].

Several interactions and synergisms between respiratory pathogens have already been described. Also infectious agents with low pathogenicity can in combination with other low pathogenic pathogens lead to high grade respiratory diseases [3]. As *P*. spp. are able to strongly proliferate under immunosuppressive circumstances in humans [4], the question arises whether this clinical pattern can also be seen in pigs. *P*. spp. have been found in humans in cases of co-infections with *Bordetella* spp. [8], *Mycoplasma* spp. [9], *Pasteurella* spp. [10], and torque tenovirus [11]. Based on these observations, the aim of the present study was the retrospective investigation of viral (porcine circovirus type 2 (PCV2), porcine reproductive and respiratory syndrome virus (PRRSV), torque teno sus virus type 1 and 2 (TTSuV1, TTSuV2)) and bacterial (*Bordetella bronchiseptica* (*B*. *b*.), *Mycoplasma hyopneumoniae* (*M*. *h*.) and *Pasteurella multocida* (*P*. *m*.)) co-infections in *P*. spp. positive lung samples of Austrian pigs with pneumonia.

## Materials and Methods

A total of 110 formalin fixed paraffin wax embedded (FFPE) lung as well as inguinal lymph node tissue samples of pigs of three different age classes (40 suckling piglets (S; < 7.5 kg body weight), 38 weaning piglets (W; 7.5–25 kg body weight), 32 fattening pigs (F; > 25 kg body weight)) were used for this retrospective study. Sixty pigs were male, 44 were female; the sex of 6 pigs was unknown. Between 1 and 3 sections per lung were evaluated. Due to the retrospective design of the study the localizations were varying. The pigs had been submitted to the Institute of Pathology and Forensic Veterinary Medicine of the University of Veterinary Medicine Vienna for routine pathological investigation during the years 2007–2013. They originated from 67 different farms (1–4 samples per farm). As the samples were collected during routinely carried out sections and were subsequently archived, no ethics committee approval was necessary. Compliance with federal law [12] and good scientific practice [13] is ensured. Haematoxylin and eosin (H&E) staining was used for assessment of histological lung lesions. The detection of *P*. spp. and PCV2 was carried out by *in situ* hybridization (ISH). *P*. *m*. and *B*. *b*. were detected using immunohistochemistry (IHC). *M*. *h*. was analysed by real time PCR, PRRSV, TTSuV1 and TTSuV2 by conventional PCR. Primer and probe sequences are presented in Table 1. *Pneumocystis* spp., *B*. *b*., *M*. *h*. and *P*. *m*. results have already been published in a related context [6].

**Table 1:** Primer and probe sequences. bp = PCR product size in base pairs, fw = forward primer, rv = reverse primer.

pathogen	Method	genomic region	bp	Sequences	references
***Pneumocystis* spp.**	ISH	18S rRNA	---	probe:5′-GGAACCCGAAGACTTTGATTTCTCATAAGATGCCGAGCGA-3′	[14]
**PCV2**	ISH	capsid protein gene	---	probe: 5′-CAGTAAATACGACCAGGACTACAATATCCGTGTAACCATG-3′	[15]
***Mycoplasma hyopneumoniae***	real time PCR	16S rRNA	126	fw: 5′-AAAGCGTCCGTAGGTTTTTT-3′; rv: 5′-TATTCCACCACTCCACTAGG-3′; probe: 5′ FAM-TCCGCTTTAGATACTGGCAAAATAGAA-TAMRA 3′	this study
**PRRSV EU+US**	PCR	ORF5	204	fw: 5′-TYCAATCAAGGCGCWGGRAC-3′; rv: 5′-TCGCCCTAATTGAATAGGTG-3′	this study
**TTSuV1**	PCR	non-coding region	303	fw: 5′-TTCGCTCGCACCACGTTTG-3′; rv: 5′-TGTCTACCGCTGGCGGCATAA-3′	this study
**TTSuV2**	PCR	non-coding region	252	fw: 5′-TCATGACAGGGTTCACCGGA-3′; rv: 5′-CGTCTGCGCACTTACTTATATACTCTA-3′	this study

### ISH for the detection of *Pneumocystis* spp. and PCV2

ISH was performed as previously described [14, 15]. The lung tissue samples were subjected to ISH for the detection of *P*. spp. For PCV2 detection, inguinal lymph node tissue samples were selected. The inguinal lymph node was chosen for PCV2 diagnostics instead of lung tissue because PCV2 has a tropism for lymphoid tissue and accumulates in large numbers in macrophages of depleted lymphoid follicles [16]. Samples confirmed as *Pneumocystis* or PCV2 positive and negative by PCR were used as controls in every run. The semiquantitative analysis for both pathogens was carried out by light microscopy and assessed using the score +++ for multiple, ++ for moderate, + for few and (+) for minimal organism or virus signals. *Pneumocystis* was scored as +++ for multiple (characterized by almost continuous lining of alveolar spaces over larger areas and frequently completely filling the alveoli), ++ for moderate (characterized by either several larger clusters of organisms which fill single alveoli or more diffuse distribution patterns with groups of organisms predominantly lining the alveolar surface), + for few (characterized by presence of *Pneumocystis* in a few foci, on the surface of alveoli, singly or in small groups), (+) for minimal signals (only scattered individual organisms), and negative (no stained organisms in the entire section). PCV2 viral load was scored as follows: +++ for multiple (abundant amounts of PCV2 nucleic acid in the cytoplasma of the majority of macrophages dispersed across the section), ++ for moderate (moderate numbers of stained cells dispersed across the section), + for few (few foci of stained cells, restricted to lymphoid follicles), (+) for minimal signals (only scattered individual stained cells), and negative (no positive cells in the entire section).

### IHC for the detection of *Bordetella bronchiseptica* and *Pasteurella multocida*

A three step indirect immunohistochemistry was performed on FFPE lung tissue samples as previously described [6]. The sections were incubated with primary polyclonal rabbit antisera specific for *B*. *b*. (dilution 1:10000), and *P*. *m*. (dilution 1:40000) and a secondary antibody biotinylated goat anti rabbit solution (ready-to-use solution; Thermo Scientific, Waltham, MA, USA). As positive controls lung samples positive in the bacteriological examination for *B*. *b*. or *P*. *m*. were used. To exclude unspecific binding, additionally a positive control was only incubated with the secondary antibody biotinylated goat anti rabbit solution. As negative controls defined negative lung samples from healthy pigs were taken. The qualitative evaluation of the slides was carried out by light microscopy and samples were categorized as positive or negative.

### DNA and RNA extraction

DNA and RNA were extracted from 5 FFPE lung tissue sections 10 μm thick (AllPrep^®^ DNA/RNA FFPE kit, Qiagen, Vienna, Austria). DNA was eluted in a volume of 60 μl buffer, RNA in 20 μl buffer. The DNA A260/280 ratio was 1.9 on average (reference range 1.7–2.0), the A260/230 ratio was 2.1 on average (reference value > 1.5). The RNA A260/280 ratio was 1.8 on average (reference range 1.8–2.0), the A260/230 ratio was 1.5 on average (reference value > 1.5).

### Real time PCR for the detection of *Mycoplasma hyopneumoniae*

The PCR reaction master mixture consisted of 10 μL TaqMan Fast Universal PCR Master Mix (2X), no AmpErase UNG (Life Technologies, Vienna, Austria), 0.5 μM of each primer, 3.125 μM probe, 2.5 μl template DNA (diluted 1:10 with distilled water) and distilled water to a total volume of 20 μl per reaction. The cycler program started with a first heat denaturation step at 95°C for 20 s, followed by 40 cycles at 95°C for 1 s and 60°C for 30 s [6]. The real time PCR had a mean efficiency of 98.904, a mean slope of -3.349, and a mean r^2^ of 0.994.

### Conventional PCRs for the detection of PRRSV, TTSuV1, and TTSuV2

The PRRSV reverse transcription (RT-) PCR of the RNA samples was carried out using the Qiagen^®^ OneStep RT-PCR kit (Qiagen, Vienna, Austria) according to the manufacturer’s instructions. RNA was diluted 1:10 prior to PCR with PCR grade water enclosed in the kit. The PCR reaction was started with a first reverse transcription step at 50°C for 30 min, followed by an initial PCR activation step at 95°°C for 15 min, 40 cycles of heat denaturation at 94°C for 30 s, primer annealing at 55°C for 30 s and DNA elongation at 72°C for 1 min. Finally, a last DNA elongation step was carried out at 72°C for 10 min. The TTSuV1 as well as the TTSuV2 PCR reaction master mixture of all PCR reactions consisted of 10 μl HotMasterMix (5Prime, Eppendorf, Vienna, Austria), 0.4 μM of each primer, 2 μl template DNA (diluted 1:5 with distilled water) and distilled water to a total volume of 25 μl. Both PCR reactions (TTSuV1 and TTSuV2) were started with a first heat denaturation step at 94°C for 2 min, followed by 40 cycles of heat denaturation at 94°C for 30 s, primer annealing at 55°C for 30 s for TTSuV1 as well as 60°C for 30 s for TTSuV2 and DNA elongation at 72°C for 1 min. Finally, a last DNA elongation step was carried out at 72°C for 10 min. An aliquot of 10 μl of each PCR product was analysed by gel electrophoresis using a 2% Tris acetate-EDTA-agarose gel. Subsequently, the agarose gel was stained with ethidium bromide and bands were visualized using the BioSens gel imaging system software (GenXpress, Wiener Neudorf, Austria). PCR products showing the expected product sizes (Table 2) were evaluated positively.

**Table 2:** Detection rates of all investigated pathogens in *Pneumocystis* positive lung samples and their relation to age groups. S = suckling piglets, W = weaning piglets, F = fattening pigs; n = number of samples.

	total prevalence (n = 110)		S (n = 40)		W (n = 38)		F (n = 32)	
	N	%	n	%	n	%	n	%
***Pneumocystis* spp.**	110	100.0	40	100.0	38	100.0	32	100.0
**PCV2**	56	50.9	18	45.0	16	42.1	22	68.8
**PRRSV**	8	7.3	3	7.5	5	13.2	0	0.0
**TTSuV1**	24	21.8	1	2.5	8	21.1	15	46.9
**TTSuV2**	53	48.2	8	20.0	18	47.4	27	84.4
***Bordetella bronchiseptica***	6	5.5	1	2.5	0	0.0	5	15.6
***Mycoplasma hyopneumoniae***	32	29.1	8	20.0	9	23.7	15	46.9
***Pasteurella multocida***	23	20.9	8	20.0	8	21.1	7	21.9

### Vector cloning for the determination of the limits of detection

Amplification products of rRNA of positive controls, which had been confirmed by sequencing by Microsynth Austria (Vienna, Austria), were cloned using the StrataClone^TM^ PCR Cloning Kit and StrataClone^TM^ Solopack Competent Cells (Agilent Technologies, La Jolla, CA, USA). Ten separate colonies were selected from the Luria-Bertani (LB) agar (Life Technologies, Vienna, Austria) and incubated over night at 37°C in LB broth (Life Technologies, Vienna, Austria). Plasmid DNA was extracted using the PureLink^®^ Quick Plasmid Miniprep Kit (Life Technologies, Vienna, Austria) and concentration was determined with a NanoVue plus spectrophotometer (GE Healthcare, Vienna, Austria). For PRRSV, the cloned DNA was used as a template for T7 and T3 polymerase (Roche, Vienna, Austria) to synthesize complementary RNA. This additional transcription step was performed with the Ribonucleosid Triphosphate Set (Roche, Vienna, Austria). A range of dilutions of the targeted genes was produced for the determination of the levels of detection (*M*. *h*. real time PCR: 4 ×10^2^/μl, PRRSV RT-PCR: 10^3^ copies/μl, TTSuV1 PCR: 10^2^ copies/μl, TTSuV2 PCR: 1 copy/μl).

### Statistical analyses

Total and age class specific prevalences were evaluated. Associations between *P*. spp. and the other pathogens and associations to various types of pneumonia are presented. Statistical analyses were undertaken using PASW 17 (SPSS, New York, NY, USA). Prevalence differences among the categories I (*P*. spp. + only viral co-infectants), II (*P*. spp. + both viral and bacterial co-infectants), III (*P*. spp. + only bacterial co-infections), and IV (*P*. spp. single infection) were analysed using χ^2^ test. Ratios of the probability of having a co-infection with *P*. spp. and any of the other pathogens were calculated using odds ratios, the respective p-values were calculated according to Altman and Bland (2011) [17]. The statistical unit was the individual sample. The level of significance was set at 5%.

## Results

The number of positive samples per pathogen for all cases as well as their relation to age class is presented in Table 2. Table 3A and 3B contain the semiquantitative score results of the *P*. spp. and PCV2 ISH. Evaluating the *P*. spp. score results, suckling piglets showed more +++ cases compared to weaning piglets and fattening pigs.

**Table 3:** a. Semiquantitative ISH results for *Pneumocystis* spp. b. Semiquantitative ISH results for PCV2. S = suckling piglets, W = weaning piglets, F = fattening pigs; n = number of samples.

**a.**								
**score**	**total prevalence (n = 110)**		**S (n = 40)**		**W (n = 38)**		**F (n = 32)**	
	**n**	**%**	**n**	**%**	**n**	**%**	**n**	**%**
+++	12	10.9	10	25.0	2	5.3	0	0.0
++	51	46.4	14	35.0	20	52.6	17	53.1
+	36	32.7	10	25.0	15	39.5	11	34.4
(+)	11	10.0	6	15.0	1	2.6	4	12.5
**b.**								
**score**	**total prevalence (n = 56)**		**S (n = 18)**		**W (n = 16)**		**F (n = 22)**	
	**n**	**%**	**n**	**%**	**n**	**%**	**n**	**%**
+++	20	35.7	8	44.4	4	25.0	8	36.4
++	21	37.5	5	27.8	7	43.8	9	40.9
+	14	25.0	4	22.2	5	31.3	5	22.7
(+)	1	1.8	1	5.6	0	0.0	0	0.0

Fifty-one % of the 110 *P*. spp. positive samples were positive for PCV2, 7% for PRRSV, 22% for TTSuV1, 48% for TTSuV2, 6% for *B*. *b*., 29% for *M*. *h*., and 21% for *P*. *m*. Co-infections and *P*. spp. single infections in total as well as their relationships to age classes and pneumonia types are presented in Table 4. An interstitial pneumonia was present in all 110 cases. Additionally, purulent bronchopneumonia (n = 38) and fibrinous/fibrino-haemorrhagic pneumonia (n = 13) occurred. Co-infections with viral pathogens were mainly related to an interstitial pneumonia, whereas bacterial respiratory agents or combinations of viral and bacterial co-infectants were present in cases with catarrhal-purulent bronchopneumonia, fibrinous or fibrino-haemorrhagic pneumonia. Lungs with *P*. spp. single infection were associated with interstitial pneumonia. The ratio of the probability of having a co-infection with *P*. spp. and any of the other pathogens was calculated on basis of odds ratios (Table 5). The exposure to *P*. spp. significantly decreased the risk of a co-infection with PRRSV in weaning piglets (p = 0.036). Tables 4 and 6 show the associations of *P*. spp. with the viral and bacterial co-infectants. In 18.2% of the *P*. spp. positive cases none of the investigated pathogens was detectable (category VI). In 29.1% of the cases a co-infection with 1 pathogen, in 28.2% with 2, in 17.3% with 3, and in 7.3% with 4 different infectious agents were observed. In 38.2% only viral (category I), in 3.6% only bacterial (category III) and in 40.0% both, viral and bacterial pathogens (category II) could be detected. Comparing the four categories, there were significant differences evaluating all samples (p <0.001) as well as on the basis of the age classes (S: p = 0.020, W: p = 0.001, F: p <0.001). The evaluation of all samples and the age class of the weaning piglets resulted in a predomination of the categories I (*P*. spp. + only viral co-infectants) and II (*P*. spp. + both viral and bacterial co-infectants). In contrast, the suckling piglets showed more samples of category I (*P*. spp. + only viral co-infectants) and category IV (*P*. spp. single infection). In the group of fattening pigs, category II (*P*. spp. + both viral and bacterial co-infectants) predominated.

**Table 4:** Co-infections and *Pneumocystis* spp. single infections in total as well as their relationships to age classes and pneumonia types. S = suckling piglets, W = weaning piglets, F = fattening pigs; category I: only viral co-infectants, category II: both viral and bacterial co-infectants, category III: only bacterial co-infectants, category IV: *Pneumocystis* spp. single infection.

categories	co-infectants	total (n =		age class						pneumonia type					
		110)		S (n = 40)		W (n = 38)		F (n = 32)		interstitial (n = 110)		purulent (n = 38)		fibrinous/fibrino-haemorrhagic (n = 13)	
		n	%	n	%	n	%	n	%	n	%	n	%	n	%
**category I**	**PCV2**	14	13.7	10	25.0	3	7.9	1	3.1	14	13.7	2	5.3	0	0.0
	**PRRSV**	2	1.8	0	0.0	2	5.3	0	0.0	2	1.8	0	0.0	0	0.0
	**TTSuV**	12	10.9	2	5.0	5	13.2	5	15.6	12	10.9	1	2.6	0	0.0
	**PCV2 + PRRSV**	2	1.8	1	2.5	1	2.6	0	0.0	2	1.8	1	2.6	0	0.0
	**PCV2 + TTSuV**	11	10.0	2	5.0	6	15.8	3	9.4	11	10.0	1	2.6	1	7.7
	**PRRSV + TTSuV**	1	0.9	0	0.0	1	2.6	0	0.0	1	0.9	1	2.6	0	0.0
**category II**	**PCV2 *+ M*. *h*.**	5	4.5	1	2.5	2	5.3	2	6.3	5	4.5	3	7.9	0	0.0
	**PCV2 *+ P*. *m*.**	1	0.9	0	0.0	0	0.0	1	3.1	1	0.9	1	2.6	0	0.0
	**TTSuV *+ B*. *b*.**	1	0.9	0	0.0	0	0.0	1	3.1	1	0.9	0	0.0	1	7.7
	**TTSuV *+ M*. *h*.**	5	4.5	1	2.5	1	2.6	3	9.4	5	4.5	4	10.5	0	0.0
	**TTSuV *+ P*. *m*.**	5	4.5	2	5.0	3	7.9	0	0.0	5	4.5	4	10.5	1	7.7
	**PCV2 + TTSuV + *M*. *h*.**	12	10.9	1	2.5	2	5.3	9	28.1	12	10.9	7	18.4	1	7.7
	**PCV2 + TTSuV + *P*. *m*.**	1	0.9	0	0.0	0	0.0	1	3.1	1	0.9	0	0.0	1	7.7
	**PRRSV + TTSuV + *P*. *m*.**	1	0.9	0	0.0	1	2.6	0	0.0	1	0.9	0	0.0	0	0.0
	**PCV2 + *M*. *h*. + *P*. *m*.**	2	1.8	2	5.0	0	0.0	0	0.0	2	1.8	0	0.0	2	15.4
	**PRRSV + *M*. *h*. + *P*. *m*.**	1	0.9	1	2.5	0	0.0	0	0.0	1	0.9	1	2.6	0	0.0
	**TTSuV + *B*. *b*. + *P*. *m*.**	1	0.9	1	2.5	0	0.0	0	0.0	1	0.9	0	0.0	0	0.0
	**TTSuV + *M*. *h*. + *P*. *m*.**	1	0.9	0	0.0	1	2.6	0	0.0	1	0.9	1	2.6	0	0.0
	**PCV2 + PRRSV + *M*. *h*. + *P*. *m*.**	1	0.9	1	2.5	0	0.0	0	0.0	1	0.9	1	2.6	0	0.0
	**PCV2 + TTSuV + *B*. *b*. + *P*. *m*.**	4	3.6	0	0.0	0	0.0	4	12.5	4	3.6	1	2.6	3	23.1
	**PCV2 + TTSuV + *M*. *h*. + *P*. *m*.**	3	2.7	0	0.0	2	5.3	1	3.1	3	2.7	2	5.3	1	7.7
**category III**	***M*. *h*.**	2	1.8	1	2.5	1	2.6	0	0.0	2	1.8	1	2.6	0	0.0
	***P*. *m*.**	2	1.8	1	2.5	1	2.6	0	0.0	2	1.8	1	2.6	1	7.7
**category IV**		20	18.2	13	32.5	6	15.8	1	3.1	20	18.2	5	13.2	1	7.7

**Table 5:** Odds ratios of *Pneumocystis* spp. positive cases for co-infections with the other pathogens. S = suckling piglets, W = weaning piglets, F = fattening pigs; n = number of samples; OR = odds ratio, CI = confidence interval, LL = lower level, UL = upper level; p = level of significance; *italic* = p ≤ 0.05.

	all samples (n = 110)				S (n = 40)				W (n = 38)				F (n = 32)			
	OR	95% CI LL	95% CI UL	p	OR	95% CI LL	95% CI UL	p	OR	95% CI LL	95% CI UL	p	OR	95% CI LL	95% CI UL	p
**PCV2**	1.206	0.707	2.058	0.502	1.391	0.512	3.777	0.528	1.000	0.402	2.486	1.000	1.722	0.635	4.538	0.275
**PRRSV**	0.476	0.193	1.174	0.107	n. a.	n. a.	n. a.	n. a.	0.291	0.092	0.925	*0*.*036*	n. a.	n. a.	n. a.	n. a.
**TTSuV1**	0.965	0.508	1.833	0.920	0.684	0.041	11.437	0.804	1.181	0.381	3.662	0.786	1.379	0.541	3.515	0.511
**TTSuV2**	0.862	0.506	1.471	0.598	1.100	0.318	3.810	0.889	1.000	0.406	2.461	1.000	1.519	0.454	5.079	0.508
***Bordetella bronchiseptica***	1.471	0.403	5.367	0.571	0.667	0.040	11.139	0.790	n. a.	n. a.	n. a.	n. a.	3.611	0.652	19.999	0.142
***Mycoplasma hyopneumoniae***	0.869	0.487	1.551	0.648	6.500	0.763	55.373	0.086	0.762	0.273	2.123	0.616	0.762	0.302	1.925	0.577
***Pasteurella multocida***	0.857	0.451	1.628	0.650	0.714	0.224	2.274	0.581	0.747	0.258	2.161	0.603	1.155	0.369	3.611	0.816
**viral co-infactants**	1.105	0.636	1.921	0.736	1.425	0.501	4.052	0.517	1.000	0.406	2.461	1.000	0.946	0.340	2.630	0.922
**viral and bacterial co-infectants**	0.904	0.526	1.554	0.728	1.467	0.439	4.900	0.545	0.635	0.248	1.624	0.349	1.558	0.590	4.119	0.378
**bacterial co-infectants**	0.629	0.172	2.295	0.493	0.658	0.087	4.979	0.698	1.000	0.133	7.491	1.000	n. a.	n. a.	n. a.	n. a.

**Table 6:** Association of *Pneumocystis* spp. with the other viral and bacterial co-infectants. no. = number; (+) = minimal, + = few, ++ = moderate, +++ = multiple organisms; numbers in brackets = numbers of cases.

	no. of co-infectants	only viral co-infectants (category I)	both viral and bacterial co-infectants (category II)	only bacterial co-infectants (category III)
*Pneumocystis* spp. +++ cases: 7/12 cases with co-infections	1	**3 cases:** PCV2++ (2); TTSuV (1)	---	**1 case:** *M*. *h*.
	2	**1 case:** PCV2+++/TTSuV	---	---
	3	---	**2 cases:** PCV2+/TTSuV/*M*. *h*.(1); PRRSV/TTSuV/*P*. *m*.(1)	---
*Pneumocystis* spp. ++ cases: 45/51 cases with co-infections	1	**13 cases:** PCV2++ (3);PCV2+ (1); PRRSV (1); TTSuV(8)	---	**1 case:** *P*. *m*.
	2	**7 cases:** PCV2++/PRRSV (1); PCV2+++/TTSuV (2);PCV2+/TTSuV (3); PRRSV/TTSuV (1)	**9 cases:** PCV2+++/*M*. *h*.(1);PCV2++/*M*. *h*.(1);PCV2+/*M*. *h*.(2);PCV2++/*P*. *m*.(1);TTSuV/*M*. *h*.(2);TTSuV/*P*. *m*.(2)	---
	3	---	**11 cases:** PCV2+++/TTSuV/*M*. *h*.(3);PCV2++/TTSuV/*M*. *h*.(1);PCV2+/TTSuV/*M*. *h*.(2);PCV2+++/TTSuV/*P*. *m*.(1);PCV2+++/*M*. *h*./*P*. *m*.(2); PRRSV/*M*. *h*./*P*. *m*.(1);TTSuV/*B*. *b*./*P*. *m*.(1)	---
	4	---	**4 cases:** PCV2+++/PRRSV/*M*. *h*./*P*. *m*.(1);PCV2++/TTSuV/*B*. *b*./*P*. *m*.(1);PCV2++/TTSuV/*M*. *h*./*P*. *m*.(2)	---
*Pneumocystis* spp. + cases: 29/36 cases with co-infections	1	**7 cases:** PCV2++ (1);PCV2+ (2);PCV2 (+) (1); PRRSV (1);TTSuV (2)	---	**2 cases:** *M*. *h*.(1);*P*. *m*.(1)
	2	**5 cases:** PCV2+++/PRRSV (1); PCV2+++/TTSuV (1);PCV2++/TTSuV (1);PCV2+/TTSuV (2)	**8 cases:** PCV2++/*M*. *h*.(1);TTSuV/*B*. *b*.(1);TTSuV/*M*. *h*.(3);TTSuV/*P*. *m*.(3)	---
	3	---	**4 cases:** PCV2+++/TTSuV/*M*. *h*.(1);PCV2++/TTSuV/*M*. *h*.(2);TTSuV/*M*. *h*./*P*. *m*.(1)	---
	4	---	**3 cases:** PCV2++/TTSuV/*B*. *b*./*P*. *m*.(1);PCV2+/TTSuV/*B*. *b*./*P*. *m*.(1);PCV2+++/TTSuV/*M*. *h*./*P*. *m*.(1)	---
*Pneumocystis* spp.(+) cases: 9/11 cases with co-infections	1	**5 cases:** PCV2+++ (3);PCV2++ (1); TTSuV (1)	---	---
	2	**1 case:** PCV2+++/TTSuV	---	---
	3	---	**2 cases:** PCV2+++/TTSuV/*M*. *h*.(1);PCV2++/TTSuV/*M*. *h*.(1)	---
	4	---	**1 case:** PCV2++/TTSuV/*B*. *b*./*P*. *m*.	---
**Total: 90/110 (81.8%) cases with co-infections**		**42 cases** (38.2%; 1:28 (25.5%); **2 co-infectants:**14 (12.7%))	**44 cases** (40.0%; **2:**17 (15.5%); **3:**19 (17.3%); **4 co-infectants:**8 (7.2%))	**4 cases** (3.6%; **1 co-infectant:**4 (3.6%))

## Discussion

The aim of the present study was the examination of possible associations between *P*. spp. and various viral and bacterial lung pathogens in pigs with pneumonia. As this was a retrospective study, only potential interactions between several infectious factors were discussed. Non-infectious factors as e. g. environmental or pig-specific factors except of the pigs’ age were not considered. Furthermore, the investigated spectrum of pathogens was limited to PCV2, PRRSV, TTSuV1 and 2, *B*. *b*., *M*. *h*. and *P*. *m*.

In 38.2% of the *P*. spp. positive cases only viral co-infectants could be determined. Two thirds of these cases had a co-infection with only one virus, mainly with PCV2 or TTSuV. In contrast, PRRSV was rather involved in poly-virally or polymicrobially caused respiratory diseases. Co-infections with *P*. spp. and PCV2 or PRRSV have already been reported. Cavallini-Sanches et al. (2006) [18] described a rate of 28% in domestic pigs co-infected with *Pneumocystis* and PCV2, Borba et al. (2011) [19] one of 20.5% in wild boars. Kim et al. (2011) [20] showed 20.5% cases with co-infection with PCV2, 12.8% with PRRSV and 48.7% with both viruses. In human medicine, only a single case report of a patient diagnosed with AIDS and a *Pneumocystis* pneumonia co-infected with a new genotype of torque teno mini virus has been published so far [11]. The present study is the first one to deal with possible associations between TTSuV and *P*. spp. in pigs. TTSuV has been claimed to cause a mild interstitial pneumonia [21], but the role of this pathogen in PRDC and its relevance as respiratory agent of economic impact is still unclear. In the present study, 25 cases were only co-infected with *P*. spp. and TTSuV with or without involvement of various bacteria, but without any concurrent PCV2 or PRRSV infection. If both, *Pneumocystis* and TTSuV are categorized as infectious agents with low pathogenicity, they still could in combination lead to higher grade respiratory diseases.

PCV2 by itself is associated with disease only under certain circumstances, but it can be activated by various infectious and non-infectious factors [22]. For example, PRRSV infection is able to enhance PCV2 replication resulting in a more severe clinical outcome compared to single infections with either PRRSV or PCV2 [23]. In contrast, associations between porcine circovirus associated diseases (PCVAD) and TTSuV have been discussed controversially [24–27]. In the present study, 14 of 110 *P*. spp. positive cases showed a co-infection with PCV2 only. Cases with ++ or +++ PCV2 signals predominated, which is an important fact as PCV2 can also be detected in tissues of clinically healthy pigs, but–in those cases–to a very low amount [28]. Nevertheless, the probability of having a co-infection with *P*. spp. and PCV2 was not significantly increased. It can be assumed that the pathogens appear successively in the evolution of infections, but due to the retrospective study design it cannot be concluded, whether infection with *Pneumocystis* or with PCV2 is coming first. Sprague-Dawley rats have become the animal of choice for *Pneumocystis* pneumonia research because of the presence of latent *P*. *carinii* infections in many breeding lines [29]. In rats, *Pneumocystis* proliferation can easily be triggered by immunosuppression [30]. In the present study there were only 12 high grade cases. In 7 of them various co-infectants could be determined. It remains unclear which predisposing factors are necessary to activate *Pneumocystis* proliferation in pigs, but based on findings in other mammalians immunosuppression seems to be essential. In contrast, synergisms and interactions between viruses seem to be sufficient for the clinical manifestation of viral diseases and immunosuppression is only considered a further conducive factor.

Only few cases had co-infections with *P*. spp. and bacteria only and these cases showed either a *P*. spp./*M*. *h*. or a *P*. spp./*P*. *m*. co-infection. To the authors’ knowledge, co-infections with *Pneumocystis* and bacteria have only been published once in pigs and once in mice so far. Kim et al. (2011) [20] identified three *Pneumocystis* positive pigs with concurrent bacterial infection. Macy et al. (2000) [10] described a co-infection with *P*. *carinii* and *Pasteurella pneumotropica* in B-cell-deficient mice and attributed clinical disease and mortality to the *Pasteurella*-induced pneumonia, but did not rule out the possibility of an indirect role of *P*. *carinii* as predisposing or synergistic factor. Co-infections with *P*. *jirovecii* and *Mycoplasma pneumoniae* in immunocompromised patients presenting with pneumonia have been investigated, but due to the low prevalence of *Mycoplasma pneumoniae* no proof of a simultaneous occurrence could be established [9]. *B*. *b*. only was described as causative for an interstitial pneumonia which can resemble *Pneumocystis* pneumonia but not in the course of co-infections [8].

In 40% of the *P*. spp. positive cases poly-microbial co-infections could be determined. Twenty-one samples were positive for *P*. spp and TTSuV or PCV2 only combined with different bacteria, followed by 20 cases positive for *P*. spp, both PCV2 and TTSuV as well as various bacteria. Co-infections including PRRSV were only rarely observed. Viruses as PRRSV or swine influenza virus have been described to be the major primary pathogens involved in porcine respiratory disease. They interact with different bacterial pathogens and over 88% of cases with respiratory disorders are caused by co-infections [31]. Infections with PRRSV, *B*. *b*. and *P*. *m*. may interact and adversely affect respiratory tract defense mechanisms [32]. The presence of *M*. *h*. is discussed to potentiate PRRSV, but also PCV2 viral load and lung lesions [33–36]. A synergistic effect of fungi has not yet been investigated. *Pneumocystis* attaches to type 1 pneumocytes, proliferates and subsequently fills the alveoli [4]. In high-grade cases alveoli are completely occluded with fungal elements [14]. As a consequence, air exchange can be compromised within the affected lung tissue which provides the optimal environment for secondary bacterial infections. Bacterial pathogens such as *P*. *m*. or *B*. *b*. produce different toxins which provoke massive tissue damages including necroses and abscesses [3]. Significant associations between *M*. *h*. and *P*. *m*. have also been described and both pathogens have been correlated with severe lung lesions [31, 37]. *P*. spp. mainly occurred in cases of interstitial pneumonia. Even in slides with pathohistological presence of different types of pneumonia, *P*. spp. signals were limited to the parts with minor lung lesions. In the authors’ opinion either pneumocytes in those advanced stages of pneumonia are too strongly damaged for an interaction with *Pneumocystis*’ filopodia or oxygen supply is suboptimal and the fungus is suppressed.

Co-infections with viral or viral and bacterial pathogens in various combinations were observed in a high percentage of *Pneumocystis* positive cases. It has to be considered that only 12 samples had a severe *P*. spp. infection. The clinical relevance of lower graded *P*. spp. infections in pigs has not yet been investigated and because of the retrospective character of the present study it remains unclear if in those cases the fungus could have proliferated. In rats, infections with low numbers of *P*. spp. in the sense of a latent infection have already been described [38] and this phenomenon could also appear in pigs. In that case, *P*. spp. could just be present as lung commensal, but would not contribute to the onset of respiratory disease.

Interactions between pathogens are the more complex the more viruses and bacteria are involved at different time points during infection. In due consideration of the severity of lung lesions, bacteria such as *B*. *b*. and *P*. *m*. as well as more advanced *M*. *h*. infections seem to occur mainly in the sense of a secondary bacterial infection, but the initial primary infection cannot be consistently defined. Nevertheless, *Pneumocystis* proliferation seems to depend on immunosuppression, whereas viral infections can also be triggered by interactions between viruses only. *M*. *h*. has also been described to act as a primary pathogen preparing the way for other pathogens as PRRSV and swine influenza virus which produce more severe lung lesions [34] and could, for this reason, also act as a precursor for *Pneumocystis*. In human medicine, metagenomic studies and comparative genomics analyses are undertaken to better characterize the lung microbiota, because especially fungal and viral agents have only been poorly studied in respiratory tract in the past, but synergistic or antagonistic interactions most probably occur [39].The application of modern methods could help to further illuminate the position of *Pneumocystis* in the course of infection.

## References

[1] Harms PA, Halbur PG, Sorden SD. Three cases of porcine respiratory disease complex associated with porcine circovirus type 2 infection. J Swine Health Prod. 2002;10: 27–30.

[2] Fablet C, Marois C, Kuntz-Simon G, Rose N, Dorenlor V, Eono F et al Longitudinal study of respiratory infection patterns of breeding sows in five farrow-to-finish herds. Vet Microbiol. 2011;147: 329–339. 10.1016/j.vetmic.2010.07.005 20696539 PMC7117213

[3] Opriessnig T, Giménez-Lirola LG, Halbur PG. Polymicrobial respiratory disease in pigs. Anim Health Res Rev. 2011;12: 133–148. 10.1017/S1466252311000120 22152290

[4] Dei-Cas E. *Pneumocystis* infections: the iceberg? Med Mycol. 2000;38: 23–32. 11204150

[5] Aliouat-Denis CM, Chabé M, Demanche C, Aliouat EM, Viscogliosi E, Guillot J et al *Pneumocystis* species, co-evolution and pathogenic power. Infect Genet Evol. 2008;8: 708–726. 10.1016/j.meegid.2008.05.001 18565802

[6] Kureljušić B, Weissenbacher-Lang C, Nedorost N, Stixenberger D, Weissenböck H. Association between *Pneumocystis* spp. and co-infections with *Bordetella bronchiseptica*, *Mycoplasma hyopneumoniae* and *Pasteurella multocida* in Austrian pigs with pneumonia. Vet J. 2015; 10.1016/j.tvjl.2015.11.003 26654847

[7] Weissenbacher-Lang C, Nedorost N, Weissenböck H. Finding your way through *Pneumocystis* sequences in the NCBI gene database. J Eukaryot Microbiol. 2014;61: 537–555. 10.1111/jeu.12132 24966006

[8] Galeziok M, Roberts I, Passalacqua JA. *Bordetella bronchiseptica* pneumonia in a man with acquired immunodeficiency syndrome: a case report. J Med Case Rep. 2009;3: 76–78. 10.1186/1752-1947-3-76 19946552 PMC2783075

[9] Govender S, du Plessis SJ, Ocana GS, Chalkley LJ. Prevalence of *Pneumocystis jirovecii* and *Mycoplasma pneumoniae* in patients presenting with pneumonia at hospitals in Port Elizabeth. South Afr J Epidemiol Infect. 2008;23: 21–24.

[10] Macy JD Jr, Weir EC, Compton SR, Shlomchik MJ, Brownstein DG. Dual infection with *Pneumocystis carinii* and *Pasteurella pneumotropica* in B cell-deficient mice: diagnosis and therapy. Comp Med. 2000;50: 49–55. 10987669

[11] Jazaeri Farsani SM, Jebbink MF, Deijs M, Canuti M, van Dort KA, Bakker M et al Identification of a new genotype of torque teno mini virus. Virol J. 2013;10: 323–329. 10.1186/1743-422X-10-323 24171716 PMC3819664

[12] Tierversuchsgesetz (Austrian federal law for animal experiments); 2012. Available: https://www.ris.bka.gv.at/GeltendeFassung.wxe?Abfrage=Bundesnormen&Gesetzesnummer=20008142

[13] Good Scientific Practice of the University for Veterinary Medicine Vienna; 2013. Available: http://www.vetmeduni.ac.at/forschung/

[14] Binanti D, Mostegl MM, Weissenbacher-Lang C, Nedorost N, Weissenböck H. Detection of *Pneumocystis* infections by *in situ* hybridization in lung samples of Austrian pigs with interstitial pneumonia. Med Mycol. 2014;52: 196–201. 23859080 10.3109/13693786.2013.809631 PMC3812124

[15] Bukovsky C, Schmoll F, Revilla-Fernandez S, Weissenböck H. Studies on the aetiology of non-suppurative encephalitis in pigs. Vet Rec. 2007;161: 552–558. 17951563 10.1136/vr.161.16.552

[16] Sorden SD. Update on porcine circovirus and postweaning multisystemic wasting syndrome (PMWS). J Swine Health Prod. 2000;8: 133–136.

[17] Altman DG, Bland JM. How to obtain the P value from a confidence interval. BMJ. 2011;343: 1–2. 10.1136/bmj.d2304 22803193

[18] Cavallini-Sanches EM, Borba MR, Spanamberg A, Pescador C, Corbellini LG, Ravazzolo AP et al Co-infection of *Pneumocystis carinii* f. sp. *suis* and porcine circovirus-2 (PCV2) in pig lungs obtained from slaughterhouses in Southern and Midwestern regions of Brazil. J Eukaryot Microbiol. 2006;53: 92–94. 10.1111/j.1550-7408.2006.00185.x 17169081

[19] Borba MR, Sanches EM, Correa AM, Spanamberg A, de Souza LJ, Soares MP et al Immunohistochemical and ultrastructural detection of *Pneumcystis* in wild boars (Sus scrofa) co-infected with porcine circovirus type 2 (PCV2) in Southern Brazil. Med Mycol. 2011;49: 172–175. 10.3109/13693786.2010.510540 20807029

[20] Kim KS, Jung JY, Kim JH, Kang SC, Hwang EK, Park BK et al Epidemiological characteristics of pulmonary pneumocystosis and concurrent infections in pigs in Jeju Island, Korea. J Vet Sci. 2011;12: 15–19. 21368558 10.4142/jvs.2011.12.1.15 PMC3053462

[21] Krakowka S, Ellis JA. Evaluation of the effects of porcine genogroup 1 torque teno virus in gnotobiotic swine. Am J Vet Res. 2008;69: 1623–1629. 10.2460/ajvr.69.12.1623 19046010

[22] Opriessnig T, Halbur PG. Concurrent infections are important for expression of porcine circovirus associated disease. Virus Res. 2012;164: 20–32. 10.1016/j.virusres.2011.09.014 21959087 PMC7114432

[23] Rovira A, Balasch M, Segalés J, Garcia L, Plana-Durán J, Rosell C et al Experimental inoculation of conventional pigs with porcine reproductive and respiratory syndrome virus and porcine circovirus 2. J Virol. 2002;76: 3232–3239. 11884547 10.1128/JVI.76.7.3232-3239.2002 PMC136035

[24] Blomström AL, Belák S, Fossum C, Fuxler L, Wallgren P, Berg M. Studies of porcine circovirus type 2, porcine boca-like virus and torque teno virus indicate the presence of multiple viral infections in postweaning multisystemic wasting syndrome pigs. Virus Res. 2010;152: 59–64. 10.1016/j.virusres.2010.06.004 20542066

[25] Kekarainen T, Sibila M, Segalés J. Prevalence of swine torque teno virus in post-weaning multisystemic wasting syndrome (PMWS)-affected and non-PMWS-affected pigs in Spain. J Gen Virol. 2006;87: 833–837. 16528032 10.1099/vir.0.81586-0

[26] Lang C, Griessler A, Pirker E, Söllner H, Segalés J, Kekarainen T et al Detection of porcine circovirus type 2 and torque-teno-sus-virus 1 and 2 in semen of boars from an Austrian artificial insemination centre. Tieraerztl Prax. 2011;39 (G): 201–204. 22138827

[27] Lang C, Söllner H, Barz A, Ladinig A, Langhoff R, Weissenböck H et al Investigation of the prevalence of swine torque teno virus in Austria. Berl Muench Tieraerztl Wschr. 2011;124: 10–15. 21465770

[28] Quintana J, Segalés J, Rosell C, Calsamiglia M, Rodríguez-Arrioja GM, Chianini F et al Clinical and pathological observations of pigs with postweaning multisystemic wasting syndrome. Vet Rec. 2001;149: 357–361. 11594382 10.1136/vr.149.12.357

[29] Bartlett MS, Fishman JA, Queener SF, Durkin MM, Jay MA, Smith JW. New rat model of *Pneumocystis carinii* infection. J Clin Microbiol. 1988;26: 1100–1102. 3260241 10.1128/jcm.26.6.1100-1102.1988 PMC266540

[30] Boylan CJ, Current WL. Improved rat model of *Pneumocystis carinii* pneumonia: induced laboratory infections in *Pneumocystis*-free animals. Infect Immun. 1992;60: 1589–1597. 1548080 10.1128/iai.60.4.1589-1597.1992 PMC257034

[31] Choi YK, Goyal SM, Joo HS. Retrospective analysis of etiologic agents associated with respiratory diseases in pigs. Can Vet J. 2003;44: 735–737. 14524628 PMC340270

[32] Brockmeier SL, Palmer MV, Bolin SR, Rimler RB. Effects of intranasal inoculation with *Bordetella bronchiseptica*, porcine reproductive and respiratory syndrome virus, or a combination of both organisms on subsequent infection with *Pasteurella multocida* in pigs. Am J Vet Res. 2001;62: 521–525. 11327458 10.2460/ajvr.2001.62.521

[33] Cho JG, Dee SA, Deen J, Trincado C, Fano E, Jiang Y et al The impact of animal age, bacterial coinfection, and isolate pathogenicity on the shedding of porcine reproductive and respiratory syndrome virus in aerosols from experimentally infected pigs. Can J Vet Res. 2006;70: 297–301. 17042383 PMC1562537

[34] Fablet C, Marois-Créhan C, Simon G, Grasland B, Jestin A, Kobisch M et al Infectious agents associated with respiratory diseases in 125 farrow-to-finish pig herds: A cross-sectional study. Vet Microbiol. 2012a;157: 152–163. 10.1016/j.vetmic.2011.12.015 22226820

[35] Opriessnig T, Thacker EL, Yu S, Fenaux M, Meng XJ, Halbur PG. Experimental reproduction of postweaning multisystemic wasting syndrome in pigs by dual infection with *Mycoplasma hyopneumoniae* and porcine circovirus type 2. Vet Pathol. 2004;41: 624–640. 15557072 10.1354/vp.41-6-624

[36] Thacker EL, Halbur PG, Ross RF, Thanawongnuwech R, Thacker BJ. *Mycoplasma hyopneumoniae* potentiation of porcine reproductive and respiratory syndrome virus-induced pneumonia. J Clin Microbiol. 1999;37: 620–627. 9986823 10.1128/jcm.37.3.620-627.1999 PMC84495

[37] Fablet C, Marois C, Dorenlor V, Eono F, Eveno E, Jolly JP et al Bacterial pathogens associated with lung lesions in slaughter pigs from 125 herds. Res Vet Sci. 2012b;93: 627–630. 10.1016/j.rvsc.2011.11.002 22133708

[38] Weisbroth SH, Geistfeld J, Weisbroth SP, Williams B, Feldman SH, Linke MJ et al Latent *Pneumocystis carinii* infection in commercial rat colonies: comparison of inductive immunosuppressants plus histopathology, PCR, and serology as detection methods. J Clin Microbiol. 1999;37: 1441–1446. 10203502 10.1128/jcm.37.5.1441-1446.1999 PMC84796

[39] Aliouat- Denis CM, Chabé M, Delhaes L, Dei-Cas E. Aerially transmitted human fungal pathogens: What can we learn from metagenomics and comparative genomics? Rev Iberoam Micol. 2014;31: 54–61. 10.1016/j.riam.2013.10.006 24286763

